# Australian Notes

**Published:** 1910-10-29

**Authors:** 


### AUSTRALIAN NOTES.

Sickness in the Australian Bush.—What may happen to a sick man in the bush was illustrated lately in the case of the headmaster of a school in the Tallangatta district, Victoria. He had contracted diphtheria, and the nearest hospital was sixty miles away at Albury, New South Wales. The first stage of thirty miles brought the patient, by nightfall in heavy rain, to the township of Tallangatta, where the discovery was made that hotel and boarding-house keepers were forbidden by the law to take in infectious cases. The patient would have had to stay in the street while a fresh team of horses were being sought, but that the doctor came to the rescue and made him comfortable in his surgery for an hour. The second stage of the journey was begun, but when only two or three miles out the buggy capsized. For an hour the patient and the driver struggled in mud and rain to right the vehicle and succeeded. When only five miles from their destination (Albury) they took a wrong turning and went on for about seven miles. Stopping at a farmhouse the driver roused the people and learned he was not on the right road—thirteen miles still had to be traversed. It was quite dark, cold, and raining, and the farmer insisted upon the travellers partaking of his hospitality. This they were indeed glad to do for some hours, and ultimately in daylight and better weather reached the Albury Hospital.

Tropical Diseases in Australia.—Dr. Anton Benil, Director of the Tropical Diseases Hospital at Townsville, Queensland, reports that he has found a good many cases of beri-beri, but chiefly among coloured people; also instances of a scaly skin disease amongst natives of Papua. He states that ankylostomiasis manifests itself with unpleasant frequency in Queensland, he having discovered traces of the disease in every house in the quarter of Cairns known as "Sandy Gallows." A common disease among cane-cutters, called "sandworm," is a skin inflammation on the inner side of the foot; but Dr. Benil was unable to discover definite evidence of the presence of parasites. In his report the Director suggests that at the beginning and end of each cane season an examination should be made of the blood and general conditions of the cane-cutters, in order to ascertain the effect of the occupation upon European workers, and he remarks upon the great practical benefit likely to result from the research work for which the Townsville hospital offers so many opportunities.

